# Small-cell lung cancer with voltage-gated calcium channel antibody-positive paraneoplastic limbic encephalitis: a case report

**DOI:** 10.1186/1752-1947-8-119

**Published:** 2014-04-08

**Authors:** Kyoichi Kaira, Takashi Okamura, Hiroki Takahashi, Norio Horiguchi, Noriaki Sunaga, Takeshi Hisada, Masanobu Yamada

**Affiliations:** 1 Department of Medicine and Molecular Science, Graduate School of Medicine, Gunma University, Showa-machi, Maebashi, Gunma 371-8511, Japan

**Keywords:** Paraneoplastic limbic encephalitis, Small-cell lung cancer, VGCC

## Abstract

**Introduction:**

Paraneoplastic limbic encephalitis is a rare neurological syndrome and clinically characterized by cognitive dysfunction, memory impairment, seizures and psychiatric symptoms. Paraneoplastic limbic encephalitis is most frequently found in small-cell lung cancer, among various malignancies, and antineuronal antibodies are related to the autoimmune mechanism. We experienced a rare case of a patient with small-cell lung cancer with anti-voltage-gated calcium channel antibody-positive paraneoplastic limbic encephalitis.

**Case presentation:**

A 61-year-old Japanese man with a history of smoking cigarettes presented with seizure, confusion and personality change in acute onset. Brain magnetic resonance imaging showed high signal intensity on T2-weighted image in his right temporal lobe, suggestive of limbic encephalitis. A mediastinoscopy of the lymph node revealed small-cell lung carcinoma, and he was staged as having limited stage disease. Antibodies against P/Q-type and N-type voltage-gated calcium channel were positive and Hu antibody was negative. He was started on chemotherapy of carboplatin plus etoposide with concurrent thoracic radiotherapy. Neurological symptoms were gradually improved after systemic chemotherapy.

**Conclusions:**

We should be alert to the potential of malignant neoplasms associated with paraneoplastic limbic encephalitis when we examine a patient with cancer with neurological disorders such as personality change, disorientation, unconsciousness and memory loss. A clinical marker such as voltage-gated calcium channel antibody may help our diagnosis in clinical practice.

## Introduction

Paraneoplastic limbic encephalitis (PLE) is an extremely rare neurological syndrome, as the initial presentation of human malignancies. The most common neoplasms include lung (50%), breast (8%) and testicular cancer (20%) [1]. PLE is clinically characterized by cognitive dysfunction, memory impairment, seizures and psychiatric symptoms [2]. The anti-Hu antibody is the most common auto-antibody detected by PLE, and anti-voltage-gated calcium channel (VGCC) antibodies are found in patients with small-cell lung cancer (SCLC), usually with Lambert–Eaton myasthenic syndrome [3]. However, it remains unclear whether antibodies to VGCC play a pathogenic role in patients with PLE. Here, we report a case of a patient with SCLC with VGCC antibody-positive PLE.

## Case presentation

**Figure 1:**
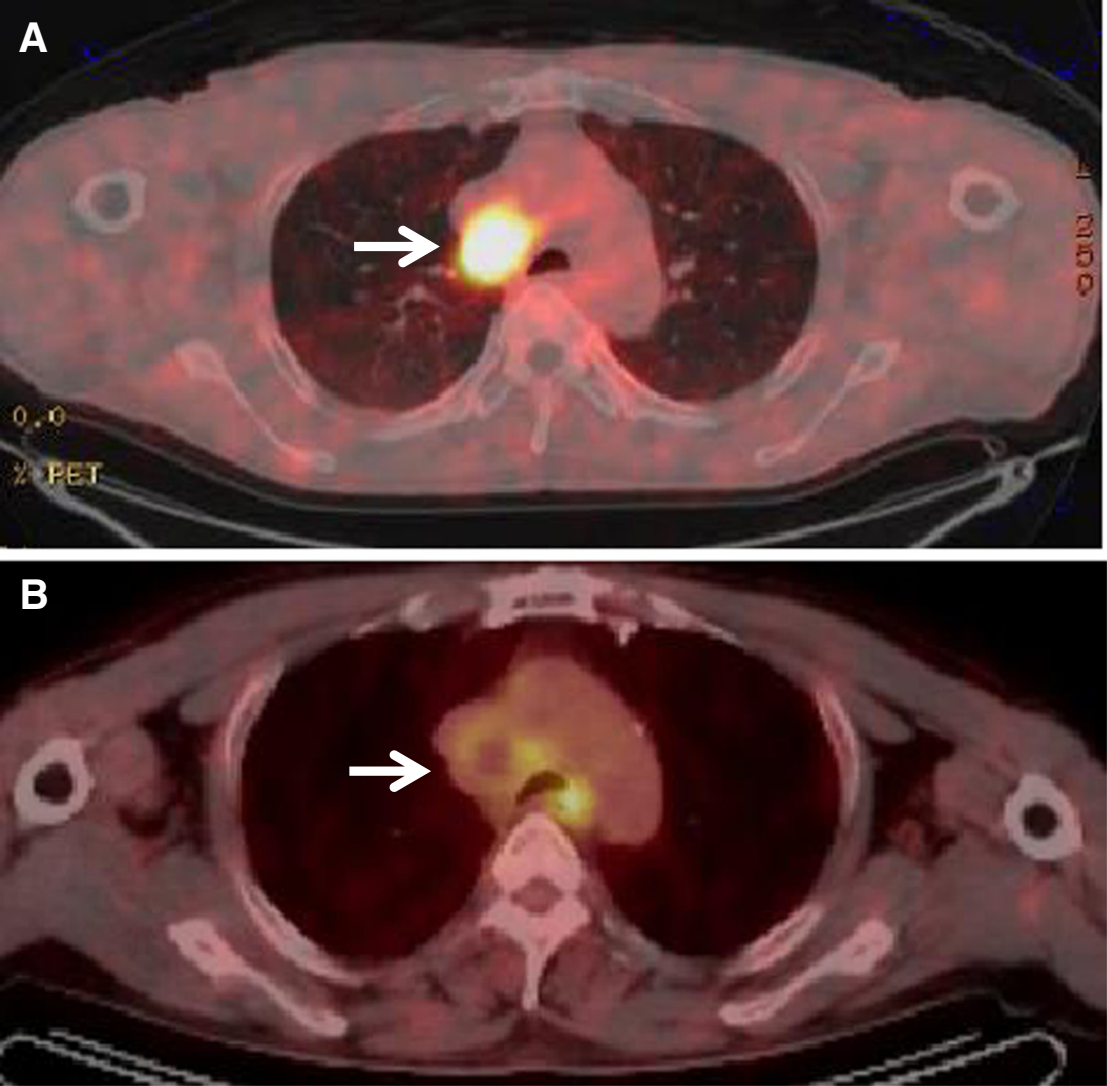
**Imaging of **^**18**^**F-fluoro-2-deoxy-D-glucose positron emission tomography at baseline and after chemotherapy. (A)** 2-[^18^F]-fluoro-2-deoxy-D-glucose positron emission tomography shows increased accumulation in the mediastinal lymph node (white arrow). **(B)** After one cycle of carboplatin plus etoposide, a positron emission tomography scan reveals a marked decrease in 2-[^18^F]-fluoro-2-deoxy-D-glucose uptake in the corresponding lesion (white arrow).

**Figure 2:**
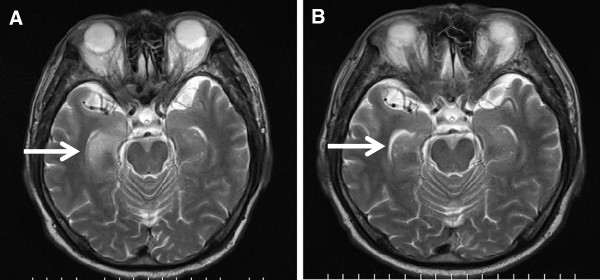
**Imaging of brain magnetic resonance imaging at baseline and after chemotherapy. (A)** Magnetic resonance imaging of the brain shows high signal intensity on T2-weighted image in the right temporal lobe (white arrow), with limbic encephalitis. **(B)** After one cycle of systemic chemotherapy, the high signal intensity was improved (white arrow).

A 61-year-old Japanese man with a history of smoking cigarettes presented with seizure, confusion and personality change in acute onset. He had no significant past medical history. On admission, a physical examination revealed consciousness disturbance with a Glasgow Coma Scale of 14, impairment of short-term memory and psychiatric symptoms. Other findings were unremarkable, including vital signs and neurological examination. A computed tomography of his thorax showed significant lymphadenopathy in the mediastinum. A 2-[^18^F]-fluoro-2-deoxy-D-glucose (^18^F-FDG) positron emission tomography (PET) scan revealed an increased accumulation in the mediastinal lymph node (Figure 1A). He continued to deteriorate following admission with progressive confusion and memory. A lumbar puncture showed a normal cerebrospinal fluid and an electroencephalogram was a normal study. However, magnetic resonance imaging (MRI) of his brain revealed abnormalities in his right temporal lobe (Figure 2A). A mediastinoscopy of the lymph node revealed small-cell lung carcinoma, and he was staged as having limited stage disease. This disease revealed a tumor status of T2N2M0. Antibodies against P/Q-type and N-type VGCC were positive and Hu antibody was negative. Antibody against voltage-gated potassium channel was within normal range. He was started on chemotherapy of carboplatin plus etoposide with concurrent thoracic radiotherapy. After one cycle of chemotherapy, his cognition and confusion markedly improved and PET showed a marked decrease of ^18^F-FDG in the lymphadenopathy (Figure 1B). A brain MRI showed that the abnormalities in his right temporal lobe had disappeared (Figure 2B). After four cycles of chemotherapy with concurrent radiotherapy, he was discharged from our institution. He had a performance status (PS) of three at diagnosis, but he improved to a PS of one after chemotherapy.

## Discussion

This is an extremely rare report of SCLC with VGCC antibody-positive PLE. A recent report had documented that antibodies to VGCC and Hu were found in 5% and 25.5%, respectively, of patients with SCLC without neurological disease (n=200), but their presence did not correlate with the extent of disease or outcome [4]. VGCC antibodies had been described to be recognized in 41% of patients with paraneoplastic cerebellar degeneration and SCLC [5]. However, patients with SCLC who have PLE have been rarely reported in the English literature, moreover, only four patients yielded a positive finding of VGCC antibody including our present case [6-8]. Although the reason for the preferential association of PLE with SCLC is unclear, several researchers hypothesize a mechanism whereby the tumor expression of brain proteins is the trigger of autoimmunity against the nervous system [1]. Patients who are anti-Hu-positive usually have multifocal neurological symptoms and SCLC is frequently associated with anti-Hu antibody. It has been reported that patients who are anti-Hu-negative with SCLC and limbic encephalitis are more likely to improve with treatment [2]. Our patient also has a negative anti-Hu antibody; therefore, his neurological symptoms may improve after systemic chemotherapy. Furthermore, recent reports had documented that some neurological symptoms were improved after systemic treatment in patients with SCLC with VGCC antibody-positive PLE [1,6-8]. However, it remains unknown whether the presence of VGCC antibody is correlated with a good response to therapy.

In the present series, a brain MRI is helpful for excluding other diseases with similar neurological disorders. It has been described that the brain MRI of patients with PLE shows a typical finding of high signal intensity on flair or T2-weighted imaging in bilateral or unilateral medial temporal lobes and/or brain stem [9,10]. Cerebrospinal fluid tapping is also necessary to exclude evidence of malignant cells or infection; this corresponds to the results of our present case. However, it is difficult for oncologists to diagnose PLE with lung cancer as an initial presentation.

## Conclusions

When we examine a patient with cancer with neurological disorders such as personality change, disorientation, unconsciousness and memory loss, we should be alert to the potential of malignant neoplasms associated with PLE. It is also important to remember that a clinical marker such as VGCC antibody may help our diagnosis in clinical practice.

## Consent

Written informed consent was obtained from the patient for the publication of this case report and its accompanying images. A copy of the written consent is available for review by the Editor-in-Chief of this journal.

## Abbreviations

18F-FDG: 2-[^18^F]-fluoro-2-deoxy-D-glucose; MRI: Magnetic resonance imaging; PET: Positron emission tomography; PLE: Paraneoplastic limbic encephalitis; PS: Performance status; SCLC: Small-cell lung cancer; VGCC: Voltage-gated calcium channel.

## Competing interests

The authors declare that they have no competing interests.

## Authors’ contributions

KK and TO operated on the patient and were major contributors in making a conception, design, acquisition of data and drafting the manuscript or revising it critically for important intellectual content. HT and NH were contributors in making a conception, design and acquisition of data. NS and TH provided advice on patient management. MY made critical revisions to the manuscript. All authors read and approved the final manuscript.
